# Exploring the validity of two memory markers for Alzheimer’s disease to detect Mild Cognitive Impairments in community‐dwelling older adults from southern Colombia

**DOI:** 10.1002/alz.091658

**Published:** 2025-01-03

**Authors:** Mario A Parra‐Rodriguez, Alfredis Gonzalez‐Hernandez, Jasmin Bonilla‐Santos, Dorian Cala, Rodrigo A Gonzalez‐Montealegre

**Affiliations:** ^1^ University of Strathclyde, Glasgow United Kingdom; ^2^ Surcolombiana University, Neiva, Huila Colombia; ^3^ Cooperative University of Colombia, Neiva, Huila Colombia

## Abstract

**Background:**

Traditional neuropsychological assessments have proved insensitive to identify the preclinical stages of Alzheimer’s disease (AD). Such a barrier has proved more challenging when assessing individuals from underrepresented populations (Parra et al., 2018; 2020; 2022). Two memory tests have been proposed as promising to identify people at risk of AD preclinically (Costa et al., 2017; Forno et al., 2022). These are the Visual Short‐Term Memory Binding Test (VSTMBT) and the Free and Cued Selective Reminding Test (FCSRT). These tests have never been used together to assess individuals from low socioeconomic backgrounds in their communities. This was the aim of this study.

**Method:**

We recruited a sample of 390 volunteers from different community settings in Southern Colombia. After applying the classical clinical criteria, 142 were classified as healthy controls (HC), and 248 received a diagnosis of mild cognitive impairment (MCI). Participants were subjected to a neuropsychological assessment using a standardized battery (Bonilla‐Santos et al., 2023) and the two memory markers for AD (i.e., VSTMBT and FCSRT). We compared groups using their background assessments and these novel memory markers. We also compared the accuracy of these tests to discriminate between cognitively unimpaired older adults and patients with MCI.

**Result:**

Patients with MCI were younger [*t*(388) = 7.2, p <0.001], had fewer years of education [t(388) = 10.42, p <0.001], and, after controlling for age and education, showed the typical amnestic multidomain profile. The VSTMBT revealed a Group by Condition interaction [*F*(1,388) = 5.60, p = 0.018], whereby MCI patients had significantly larger deficits when processing bound information than individual features. The FCSRT significantly discriminated between patients and controls being free recall the most sensitive variable. None of these outcomes changed after controlling for education, sex, and comorbidities. ROC analysis revealed an AUC of 79% and 72% for the VSTMBT and FCSRT, respectively.

**Conclusion:**

The VSTMBT and FCSRT proved sensitive to identifying older people with MCI who had not sought medical advice at the time of testing. These two screening tools are useful for detecting people at risk of AD in community settings of underserved populations.

